# FTIR Biospectroscopy Investigation on Cisplatin Cytotoxicity in Three Pairs of Sensitive and Resistant Cell Line

**Published:** 2016

**Authors:** Ensieh Farhadi, Farzad Kobarfard, Farshad H. Shirazi

**Affiliations:** a *Department of Toxicology and Pharmacology, Shahid Beheshti University of Medical Sciences, Tehran, Iran. *; b *Department of Medicinal Chemistry, Shahid Beheshti University of Medical Sciences, Tehran, Iran. *; c *Pharmaceutical Sciences Research Center, Shahid Beheshti University of Medical Sciences, Tehran, Iran.*

**Keywords:** Cisplatin Resistance, FTIR, A2780, OV2008, HTB56

## Abstract

Fourier Transformed Infrared Spectroscopy (FTIR) has extensively been used for biological applications. Cisplatin is one the most useful antineoplastic chemotherapy drugs for a variety of different human cancers. One of the clinical problems in its application, which would consequently affect the therapeutic outcome of its application, is the occurrence of resistance to this agent. In this project three different pairs of sensitive and resistant cell lines of human ovarian A2780 and its resistant pair of A2780-CP, human ovarian OV2008 and its resistant pair of C13, and finally human lung carcinoma of HTB56 and its resistant pair of HTB56-CP were grown in the laboratory under the standard procedure. Saline was exposed to control cells, whereas 1, 5 and 10 µg/mL of cisplatin was exposed to experimental cells, for 1 h. Cells were then collected and lyophilized from which spectra were taken. According to our results, we could not trigger a well-recognized cells biomolecular band at 1015 cm^-1^, being modified after exposure to cisplatin in all cell lines. On the other hand, there was a clear dose-dependent increase in protein β-sheet structure related peaks shift in resistant cell lines after exposure to cisplatin. This would probably indicate an easier protein interaction site for cisplatin in the resistant cell lines, which would probably inhibit cisplatin from binding to DNA, as the cytotoxic target. As a conclusion, FTIR biospectroscopy has proven its potency to identify the interactions, as well as the false engagement cellular sites for cisplatin in sensitive and resistant cell lines.

## Introduction

Cancer is one of the life-threatening diseases, killing many people every year. Cisplatin is one of the most effective anticancer drugs for the treatment of ovarian, breast, head and neck, and childhood leukemia (1). One of the most important factors which is limiting cisplatin therapeutic efficacy in cancer treatment is the development of resistant tumor cells (2). Whether the resistance to cisplatin is through the drug uptake, drug degradation in cells or the development of cellular proteins that detoxifies this agent is not yet well clear (3). Investigators are trying to understand the potential responsible mechanisms for the resistance to cisplatin, with the hope to overcome this problem and increase platinum based cancer chemotherapy outcome (4).

Among the different methods for the investigation on the cellular mechanisms is biospectroscopy (5). Using different spectroscopic methods in this technique, investigators are trying to make correlations between the cellular spectral alterations with the biological events. Although the application of this technique as a creditable methodology for scientific investigation is not yet well established, a very promising future is on the horizon (6). Fourier Transformed Infra-red (FTIR) spectroscopy is one the most useful spectroscopic methods used in biology. Previous publications have tried to correlate some particular FTIR spectroscopic regions with some biomolecular structures (7). Scientists have also tried to derive biological, pathological and pharmacological applications from the resulted data (8-10). Scientific challenges are not only on the interpretation of biological samples FTIR spectra, but also on the technical aspects which are used in acquiring a well reproducible, accurate and precise spectra.

In this study, we have tried to apply FTIR spectroscopy to investigate three paired human tumor cell lines, sensitive and resistance to cisplatin. The idea is to compare the spectral patterns of sensitive and resistant cell lines to each other and look for the most likely related peaks appeared, disappeared and/or altered in all three pairs of resistant vs. sensitive cell lines, and/or following the exposure to cisplatin. We are presenting data, for the first time, to support the application of FTIR spectroscopy for pharmacological investigations, as well as clues to clarify the mechanism of resistance to cisplatin in these cell lines.

## Experimental

*Cell lines*

Human ovarian carcinoma cell lines of OV2008 and A2780 (12), and their respected cisplatin resistant variants of Ov2008-CP (C13) and A2780CP, Human lung adenocarcinoma HTB56 (ATCC® HTB-56™) and its cisplatin resistant variant of HTB56-CP were obtained from the Pharmacology Lab, Ottawa Regional Cancer Centre, Ottawa, Ont., Canada. The cells were cultured following the standard methodology of the American Type Culture Collection (ATCC, Rockville, MD). Cells were cultured in DMEM/F12 medium (Gibco BRL, USA) supplied with 10% heat-inactivated fetal bovine serum in 37 ºC humidified incubator with 5% CO_2_. All cellular experiments were done in triplicate. All experiments were performed on the exponentially growing cells, after a minimum of three passages from the initial seed of frozen stock. 

*Chemicals*

Cisplatin was the clinical formulation of 1 mg/mL in 0.9% saline supplied by Horner Laboratories (Montreal, Canada). This formulation was diluted in additional 0.9% saline to the appropriate concentrations and incubated for 1 h at 37 °C before use.

*Drug Treatment of cells*

Cells were grown in 6-well plates separately and then were exposed to 1, 5, and 10 μg/mL of cisplatin in serum-free media to prevent cisplatin denaturation through the protein binding, based on the published method (12). Control cells were exposed to the equivalent volumes of sterile saline. After one-hour incubation of cells with the above solutions, media was aspirated and cells were rinsed three times with saline. Cells were collected by trypsinization gently in 2 mL of serum-added media to inhibit extra-digestion by trypsin. Cells were then collected in 15 mL cone centrifuge vials, washed twice with saline and then centrifuged (250 g for 10 min) at room temperature. The cell pellets in 1 mL saline were then obtained for analysis. The experiments were performed in triplicate for each concentration of cisplatin. 

*Freeze-drying protocol*

Cells lyophilization was performed using a Usifroio model SMH-50 instrument. Readily collected cells were transformed into specific lyophilization 10 mL vials and the initial freezing was performed at -40 °C in 2.5 h. Primary drying procedure was then conducted on these cells during 4.5 h at the same temperature at 5x10^-2^ millibar pressure. Temperature was then increased gradually up to the room temperature of 25 °C in 22 h under the same vacuum condition. Vial lead was then sealed using 7x10^-3^ millibar pressure. These vials containing freeze-dried cells were kept in refrigerator before the FTIR experiments. 

*FTIR analysis*

At the time of spectroscopy, vials containing freeze-dried cells were opened and 5 mg of cell powder were mixed with 95 mg of potassium bromide rapidly. A small amount of this mixed powder was put in the sample holder of a Perkin Elmer instrument model Spectrum one provided with a TGF detector, and analyzed using the DRIFT (Diffuse Reflectance Infra-red Fourier Transform) method. Pure potassium bromide was used as the control for background spectra. 

For each spectrum, a total of 512 scans, at 4 cm^-1^ resolution, were applied in the range of 600-4000 cm^-1^. Spectral mathematical manipulations (as the baseline corrections, smoothing, and deconvolution) and statistical parameters (such as peak frequency shifts and intensity ratios of various infrared bands) were calculated using Perkin Elmer software 1B version 3.02. 

*Statistical analysis*

Different spectral parameters were used for the comparison purposes between the control and cisplatin exposed cells, including wave numbers (cm^-1^) and absorbance ratios based on the peak height and area under the curves. Results, expressed as mean ± S.D in tables and graphs were created using nonlinear regression analysis in GraphPad PRISM^®^ Software. One-way analysis of variance (ANOVA) followed by Dunnett's post hoc test was used to assess significant differences (*p *< 0.05) between spectra resulted from different cells exposed to different concentrations of cisplatin. All the statistical analysis was performed using GraphPad PRISM^®^ version 5 Software (GraphPad Software, San Diego, CA, USA).

## Results

**Figure 1:**
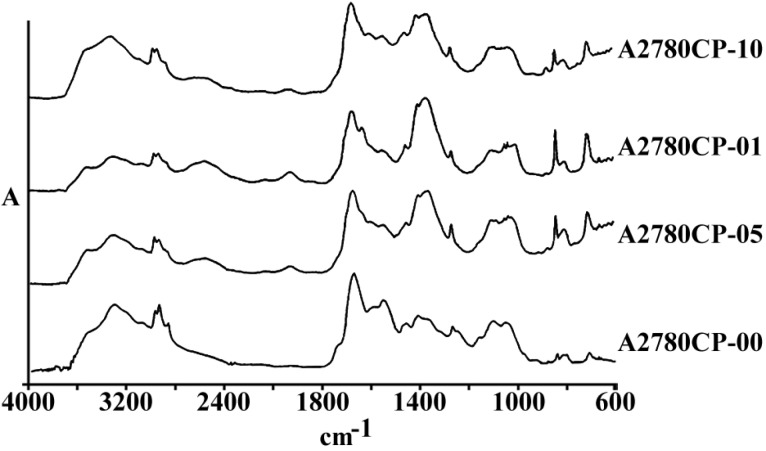
a) The general pattern of FTIR spectra of under study cell lines (refer to text for the name and specification of cell lines), and b) The FTIR spectra of Human ovarian adenocarcinomas of OV2008 (upper graphs) and its cisplatin resistant variant of A2780-CP (lower graphs) after exposure to 0, 1, 5 and 10 µg/mL of cisplatin for 1 h, in the region of 600-4000 cm^-1^

Figure 1 represents the total spectra of human ovarian adenocarcinoma A2780 and its resistant variant after 1 h exposure to 1, 5 and 10 µg/mL of cisplatin, or saline as control, for 1 h in the range of 600-4000 cm^-1^. As is shown in these spectra, there are several features related to the different cell components that are altered after the exposure of cells to cisplatin. The area of above 2000 cm^-1^ has mainly been correlated with the CH_2 _of lipid bi-layer matrix of the cells. Most of the information on the cellular proteins are coming from the amide I and II bands represented in the area of 100-1700 cm^-1^. Different features related to DNA and its backbone is absorbed around the area of 1200 cm^-1^, followed by the spectra of cellular carbohydrates in the neighboring region. Changes, if any, observed in any particular regions are discussed ahead. 

**Figure 2:**
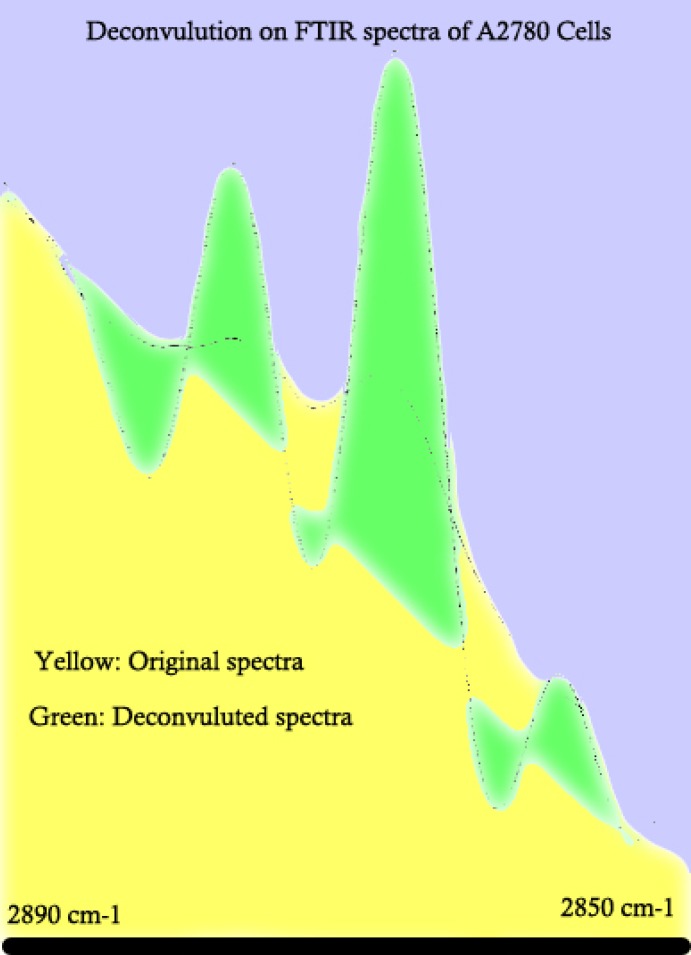
Example of FTIR spectral deconvulotion on human cell lines representing the result on human ovarian adenocarcinoma of OV2008 as one example. Deconvulotion helps to identify the peaks with a higher precision

To better understand the cellular spectral changes in different cell lines, mathematical revisions are needed. Deconvolution is a well-known technique to extrapolate different co-covered peaks from each other. Figure 2 represents a sample of mathematical deconvolution on A2780 cell line spectra to further clarify some regions of spectra for a better peak determination and comparison purposes. 

**Figure 3:**
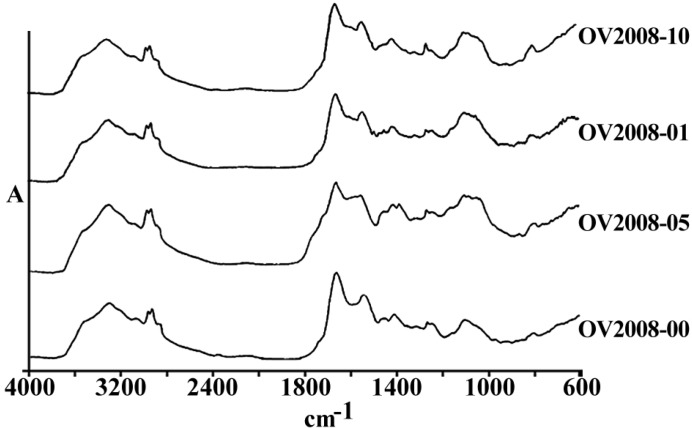
Comparison of spectral peaks shifts on four peaks corresponding to the cellular absorbance of carbohydrates, symmetrical and unsymmetrical stretching of phosphate bands in three human cell lines (upper graphs) and their cisplatin resistant variants (CPs in lower graph

**Figure 4:**
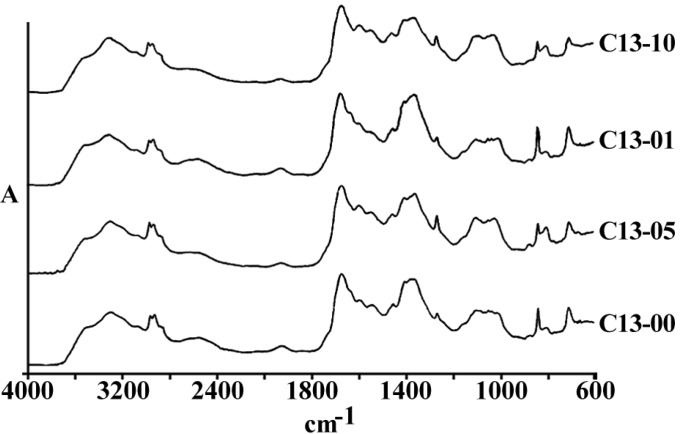
Comparison of spectral peaks shifts on four peaks corresponding to the cellular absorbance of nucleic acids and DNA bands in three human cell lines (upper graphs) and their cisplatin resistant variants (CPs in lower graph

**Figure 5:**
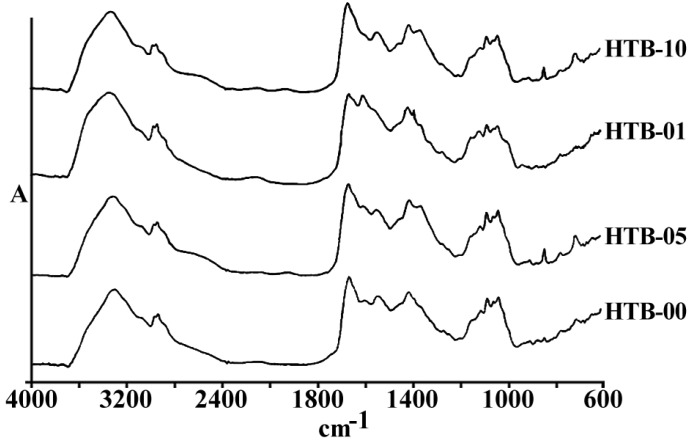
Comparison of spectral peaks shifts on four peaks corresponding to the cellular absorbance of amide bands arising from proteins in three human cell lines (upper graphs) and their cisplatin resistant variants (CPs in lower graph

**Figure 6:**
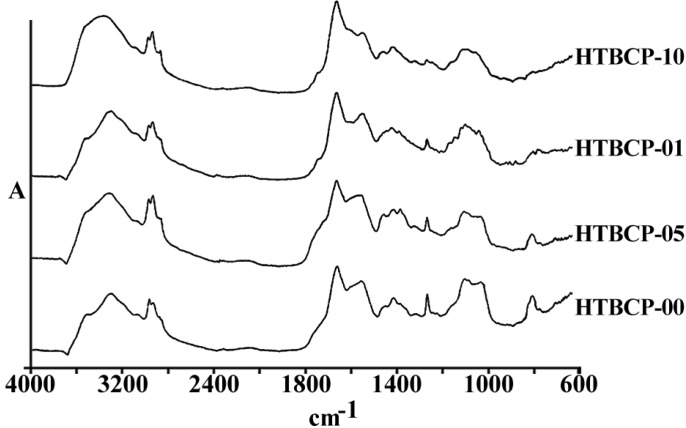
Comparison of spectral peaks shifts on four peaks corresponding to the cellular absorbance of CH and CH_2_ bands arising from cell bilayer lipid membrane matrix vibration of three human cell lines (upper graphs) and their cisplatin resistant variants (CPs in lower graph

Figures 3 and 4 are representing the alterations of spectra in the ovarian cell lines sensitive (OV2008) and sensitive (C13) to cisplatin following exposure to this drug. As are shown in these figures and are more elaborated in the discussion section the most changes are observed around 1200-1700 cm^-1^ which are more correlated with the DNA and proteins. Figures 5 and 6 are representing the spectral alterations in the sensitive and resistance pair of lung cells alone (concentrations 0) or after exposure to the different concentrations of cisplatin. Again, most of the variations are seen in the same area of 1200-1700 cm^-1^ coming from cellular DNA and proteins. As are shown in these figures, most of the intracellular variations as well as the sensitivity to cisplatin parameters in these cell lines should be found among the cellular DNA or proteins. Not all of these regions and their alterations have yet been well defined for the cells; however, those changes which are better understood based on the previous publications are elaborated and the biological meanings of these changes are discussed in the following section. 

## Discussion

Cancer chemotherapy success is to a high extent related to the cellular response to the selected chemotherapeutic agent. Time has a major role in the chemotherapy outcome, although it may take months before the physician can estimate the tumor response to the selected medication. A fast and reliable technique to understand the sensitivity or resistance of a tumor type to different cancer chemotherapy agents will save the critical time and will benefit patients. Fourier Transform Infrared Spectroscopy (FTIR) is one of the most promising techniques to distinguish biological variations among different samples. Simplicity, prompt response, sensitivity and subject independence properties hallmark this technique among other conventional diagnostic methods. Although it is in the beginning and discovering steps, its advantages on cancer detection have widely been examined (13) and new applications are coming by the time.

We have aimed to try this technique for the estimation of cancer cells sensitivity to cisplatin cytotoxicity as a model for its application in chemotherapy outcome prediction use. Cisplatin is one of the most widely used cancer chemotherapeutic agents in clinics, and many sensitive or resistance cell lines to this agent are recognized and well established. Three pairs of the same origin, but cisplatin sensitive and resistant variant cell lines, were selected in this study and exposed to the clinically relevant doses of cisplatin for a double period of clinically relevant half life of this drug. Cells were then collected, lyophilized and examined under FTIR instrument to look for a well discriminative peak to be consistent with sensitivity or resistance of the cell line to cisplatin. Lyophilization has been introduced to the collected cells to set up a repeatable unique method of cell preparation and long term preservation to be useful for biospectroscopy, which will also eliminate the large proportion of cellular and intracellular water with its related large interfering peak which mask many spectroscopic peaks from various cellular biomolecules in FTIR spectroscopy. A good and acceptable reproducibility in cell spectra have also been acquired using the lyophilized cells instead of other sample drying or H_2_O/D_2_O substitute techniques (data not shown).

Zendehdel *et al* (14) have also used FTIR biospectroscopy to distinguish between one human sensitive to cisplatin ovarian cell line with two resistant variant cell lines using PCA analysis. They have looked at bands in four different segments of spectra in the ranges of 1000-1500, 1500-2000, 2000-2500 and 2500-3000 cm^-1^. Although no discriminative peaks have been found in the two segments of 1500-2000 and 2500-3000, bands at 1240, 1330, 1380, and 1407 cm^-1^ were clearly recognizing sensitive from resistant cell lines. As is shown in Figure 5, a peak at 1645 cm^-1^ arising from protein β-sheets is clearly decreased after the exposure of resistance cell line of HTB-56 after the exposure to cisplatin indicating the cisplatin interaction with a protein which affects its conformational orientation.

The significance of the present work in comparison to the previous ones is that three pairs of well-established and confirmed cisplatin resistant and sensitive cell lines are examined using FTIR spectroscopy with and without exposure to this drug. Using such a unique protocol, we were hoping on not only to test and prove the application of FTIR biospectroscopy for these purposes in more detail, but also to collect hint for the cisplatin sensitivity/resistance determinants of cellular biomolecular sites with the study of peaks shifts after exposure to the different concentrations of cisplatin in comparison to control. 

Among the different tested regions, peaks at 1015 cm^-1^ have disappeared or shifted to a lower wave number after exposure to cisplatin in almost all resistance cell lines compared to their matched sensitive variants (Figure 6). Although this FTIR band has been correlated to the Cβ-(CH_3_)_2_ symmetric stretching in aliphatic compounds, it has not yet been well recognized in biological structures of cell lines (15). In any case, lower wave numbers correlate to higher frequencies and more required energy for the vibration. Observed shift of this peak to a lower wave number after the cell exposure to cisplatin in our experiments might be related to the attachment of the heavier and covalently bound cisplatin to the corresponding group of the respected biomolecules in these cells as one of the major cisplatin candidate sites, which need to be identified in future.

The human ovarian adenocarcinoma A2780 is also presenting two unique peaks appearances for which none of other cell lines are incorporating. The peak at 1080 cm^-1^ in cisplatin sensitive A2780 cell line is shown at 1100 in other cell lines, and the peak at 1628 cm^-1^ in cisplatin resistance A2780-CP cell line is presented at 1635 in other cell lines. The peak around 1080 to 1100 cm^-1^ is correlated to the symmetric stretching of PO_2_ groups in cellular phospholipids. Presence of this peak in a lower wave numbers in A2780 cell line compared to the other cancerous cells studied in this project might indicate a tighter phospholipid structure of the cell membrane in these cells. This unique structural feature for A2780 cells has been mentioned in previous publications of Taylor *et al.* when they were investigating the resistance mechanisms of this cell line to cisplatin (16), analyzing the cell membrane constituent of ovarian cell lines. The peak around 1625 to 1535 cm^-1^, on the other hand, has been correlated to the total cellular protein β-sheet structures (17). The fact that this peak is presented in a lower wave number in the resistant variant of A2780 cells means a higher energy (corresponding to higher frequency) is needed to vibrate these cellular biomolecules in this cell line. This phenomenon might be interpreted as cisplatin covalent binding to the β-sheet structure proteins instead of the α-structured DNA. Such an event might well preserve these cells from the cisplatin active site in other cell lines, which is shown to be the covalent bind to DNA, introducing a new hypothesis for the mechanism of resistance to cisplatin in this cell line.

## Conclusion

As a conclusion, our work has further proved the application of FTIR spectroscopy in biological discoveries. Our unique protocol of FTIR biospectroscopy on three sensitive and resistance cell lines to cisplatin and after exposure to the different concentrations of this agent has presented some candidate biomolecular sites for the action, as well as resistance to this drug in these cell lines, to be further investigated in detail in future works. Here, we have presented clues to support a new hypothesis that resistance to cisplatin might well be related to the engagement of this agent to β-sheet structures of proteins (indicating with peak at 1625 cm^-1^), cellular phospholipids (indicating with peak at 1080 cm^-1^) and a not yet known biomolecule indicated at 1015 cm^-1^, instead of cisplatin target site on the α-helical structure of DNA. More investigations are ongoing to further explore this finding.

## References

[1] Petrelli F, Zaniboni A, Coinu A, Cabiddu M, Ghilardi M, Sgroi G, Barni S (2013). Cisplatin or not in advanced gastric cancer: A systematic review and meta-analysis. PLoS One.

[2] Itamochi H, Nishimura M, Oumi N, Kato M, Oishi T, Shimada M, Sato S, Naniwa J, Sato S, Kudoh A, Kigawa J, Harada T (2014). Checkpoint kinase inhibitor AZD7762 overcomes cisplatin resistance in clear cell carcinoma of the ovary. Int J Gynecol Cancer.

[3] Xue X, Hall MD, Zhang Q, Wang PC, Gottesman MM, Liang XJ (2013). Nanoscale drug delivery platforms overcome platinum-based resistance in cancer cells due to abnormal membrane protein trafficking. ACS Nano.

[4] Boulikas T, Vougiouka M (2003). Cisplatin and platinum drugs at the molecular level. Oncol Rep.

[5] Shirazi FH, Vakili N, Abdi K, Farhadi E, Rahimi F (2007). Fourier-transform infrared spectroscopic comparison of normal and malignant cervical tissue cervical tissue. Iran J Pharm Res.

[6] Trevisan J, Angelov PP, Carmichael PL, Scott AD and  Martin FL (2012). Extracting biological information with computational analysis of Fourier-transform infrared (FTIR) biospectroscopy datasets current practices to future perspectives. Analyst.

[7] Bellisola G, Sorio C (2012). Infrared spectroscopy and microscopy in cancer research and diagnosis. Am J Cancer Res.

[8] Sahu RK, Mordechai S (2010). Spectral signatures of colonic malignancies in the mid-infrared region: from basic research to clinical applicability. Future Oncol.

[9] Mackanos MA, Contag CH (2010). Fiber-optic probes enable cancer detection with FTIR spectroscopy. Trends Biotechnol.

[10] Ashtarinezhad A, Shirazi FH, Vatanpour H, Mohammadzadehasl B, Panahyab A, Nakhjavani M (2015). FTIR-microspectroscopy detection of metronidazole teratogenic effects on mice fetus. Iran J Pharm Res.

[11] Korch C, Spillman MA, Jackson TA, Jacobsen BM, Murphy SK, Lessey BA, Jordan VC, Bradford AP (2012). DNA profiling analysis of endometrial and ovarian cell lines reveals misidentification, redundancy and contamination. Gynecol Oncol.

[12] Baribeau S, Chaudhry P, Parent S, Asselin E (2014). Resveratrol Inhibits Cisplatin-Induced Epithelial-to-Mesenchymal Transition in Ovarian Cancer Cell Lines. PLoS One.

[13] Zendehdel R, Shirazi FH (2015). Discrimination of human cell lines by infrared spectroscopy and mathematical modeling. Iran J Pharm Res.

[14] Zendehdel R, Masoudi-Nejad A, Mohammadzadeh J, Shirazi FH (2012). Cisplatin resistant patterns in ovarian cell lines using FTIR and principal component analysis. Iran J Pharm Res.

[15] Ravikumar B, Ramaswamy S, Pandiarajan S (2012). FTIR and laser raman spectral analysis of crystalline DL-valinium dihydrogen phosphate. Int J Eng SciTechnology.

[16] Taylor KD, Goel R, Shirazi FH, Molepo M, Popovic P, Stewart DJ, Wong PT (1995). Pressure tuning infrared spectroscopic study of cisplatin-induced structural changes in a phosphatidylserine model membrane. Br J Cancer.

[17] Zendehdel R, Masoudi-Nejad A, Shirazi FH (2012). Patterns prediction of chemotherapy sensitivity in cancer cell lines using FTIR spectrum, neural network and principal components analysis. Iran J Pharm Res.

